# LCRAnnotationsDB: a database of low complexity regions functional and structural annotations

**DOI:** 10.1186/s12864-024-10960-5

**Published:** 2024-12-27

**Authors:** Joanna Ziemska-Legiecka, Patryk Jarnot, Sylwia Szymańska, Dagmara Błaszczyk, Alicja Staśczak, Hanna Langer-Macioł, Kinga Lucińska, Karolina Widzisz, Aleksandra Janas, Hanna Słowik, Wiktoria Śliwińska, Aleksandra Gruca, Marcin Grynberg

**Affiliations:** 1 Institute of Biochemistry and Biophysics, Polish Academy of Sciences, Warsaw, 02-106 Poland; 2 Department of Computer Networks and Systems, Silesian University of Technology, Gliwice, 44-100 Poland; 3 Malopolska Centre of Biotechnology, Jagiellonian University, Kraków, 30-387 Poland; 4 Biotechnology Center, Silesian University of Technology, Gliwice, 44-100 Poland; 5 Department of Systems Biology and Engineering, Faculty of Automatic Control, Electronics and Computer Science, Silesian University of Technology, Gliwice, 44-100 Poland; 6 Faculty of Automatic Control, Electronics and Computer Science, Silesian University of Technology, Gliwice, 44-100 Poland; 7 Department of Graphics, Computer Vision and Digital Systems, Silesian University of Technology, Gliwice, 44-100 Poland; 8 Department of Clinical and Molecular Genetics, Maria Sklodowska-Curie National Research Institute of Oncology, Gliwice Branch, Gliwice, 44-100 Poland

**Keywords:** Protein, LCR, LCD, Low complexity regions, Annotation, Database, Integration, Function, Structure

## Abstract

Low Complexity Regions (LCRs) are segments of proteins with a low diversity of amino acid composition. These regions play important roles in proteins. However, annotations describing these functions are dispersed across databases and scientific literature. LCRAnnotationsDB aims to consolidate knowledge about LCRs and store relevant annotations in a single place. To unify redundant annotations, we assigned them categories based on similarity in function, protein structure, and biological process. Categories are organized hierarchically by linking them to Gene Ontology terms. The LCRAnnotationsDB database can be accessed at https://lcrannotdb.lcr-lab.org/.

**Supplementary Information:**

The online version contains supplementary material available at 10.1186/s12864-024-10960-5.

## Background

For a long time, Low Complexity Regions (LCRs) were considered non-functional and disordered fragments of proteins [1]. Consequently, LCRs were filtered out during analyses, resulting in a lack of resources that could provide systematized knowledge about their functions. In recent years, however, researchers have shown that many LCRs play important roles in proteins and have annotated their functions and structures in general protein databases [2, 3]. Despite these annotations, relevant data are scattered across various resources and scientific articles. Additionally, each database uses distinct identifiers and terms in protein annotations for the same characteristics. As a result, to obtain unified information about proteins, researchers use Gene Ontology (GO) to describe their functions in a standardized manner and automatically analyze large protein datasets using machine learning models [4]. Currently, GO is the most common source for automated functional annotation of protein sequences. However, this approach cannot be easily applied to the functional analysis of LCRs, because they rarely play a global function in proteins and are usually short fragments of the protein sequence.

Proteins are usually grouped by their functional and evolutionary relationships into families, which can be analyzed and stored in databases such as InterPro [5]. Additionally, we can group proteins based on their similar functions, even if these proteins do not have similar sequences. Here we focus on specific short fragments, LCRs only, that determine specific local functions, that can influence the biological function of a protein. For example, a group of proteins with fragments rich in arginine binds to RNA and supports various functions of globular proteins [6, 7]. These regions can be homorepeats of arginine, as seen in the REV protein, or more irregular LCRs, as found in the antitermination protein N [8–10]. In both cases, these regions bind RNA and overlap with LCRs. LCRs are characterized by a low diversity of amino acids and may play important functional roles in proteins. Another example is liquid-liquid phase separation (LLPS) proteins that contain LCRs. LCRs in these proteins are located in crucial regions required for binding nucleotides or proteins and for phase separation [11–13]. For instance, the nucleoporin NSP1 contains an FG-rich region consisting of several LCRs. This region forms a hydrogel through interactions between repeats in LCRs [11].

In this work, we introduce LCRAnnotationsDB, which enables functional analysis of LCRs. Four services with data partially overlapping with our database exist: HRaP, LCR-eXXXplorer, LCD-Composer, and PlaToLoCo. The HRaP database contains homorepeats and predicted disordered patterns [14], providing information only about a small subset of LCRs without functional annotation, except for GOs. Additionally, it is unclear how frequently the information in this database is updated. LCR-eXXXplorer provides information on LCRs identified using the CAST [15] and SEG [16] methods, enriched with data on GO terms assigned to proteins, disordered regions identified using the IUPred [17] and ANCHOR predictors, and functional annotations from the UniProt/SwissProt database. While LCR-eXXXplorer is a valuable resource, it is not updated regularly, and the functional LCR annotations are limited. In contrast, LCRAnnotationsDB incorporates annotations from nine additional external databases making our database the most comprehensive source of information on LCR functions. The LCD-Composer website allows searching for LCRs in proteins rich in a selected set of amino acids [18]. It does not provide additional functional annotations, except for GO terms assigned to proteins. The LCD-Composer results are available for download as TSV files, and the service does not offer any visualization of the results. HRaP, LCR-eXXXplorer, and LCD-Composer provide GO terms annotating the whole protein sequence, while Gene Ontology annotations available in LCRAnnotationsDB are assigned to LCR fragments only, ensuring that the functional information is specific to the particular low complexity fragment. PlaToLoCo identifies LCRs using various state-of-the-art methods and provides annotations of Pfam domains, predicted transmembrane regions, and signal peptides [19]. The functional information on LCRs provided by PlaToLoCo is limited, as its main aim is to integrate information from different tools for LCR discovery. None of these services include *unified* functional and structural information from a large number of publicly available protein databases.

To collect and systematize LCR annotations, we created LCRAnnotationsDB. This database contains unified information about LCRs and their functions, integrated from publicly available sources, making it unique among other resources. We introduced a category system that groups similar annotations from different source databases. This method and outcome will help scientists describe LCRs of interest that are not annotated in canonical databases such as InterPro [5], SMART [20], or DisProt [21]. Researchers can use this information to discover new functions of LCRs and proteins by sequence similarity inference. LCRAnnotationsDB allows easy provides to information about LCR functions and structures for analysis.

## Construction and content

### Construction

**Fig. 1:**
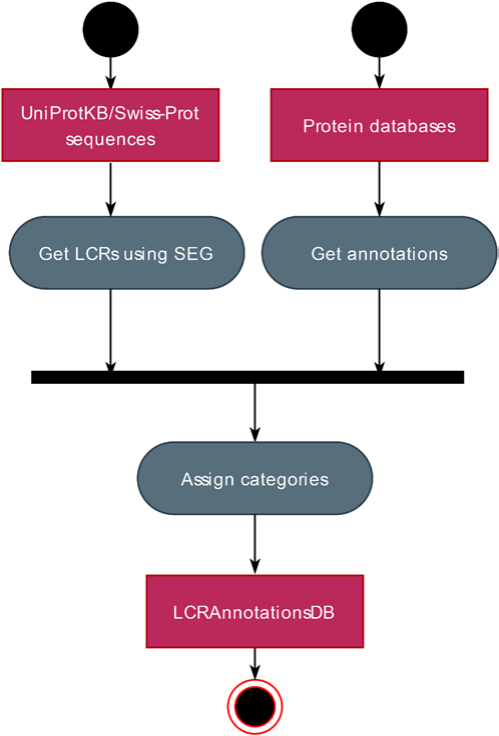
Workflow summarizing the creation of LCRAnnotationsDB. Pink boxes contain input data for processes in grey boxes. Black dots indicate the beginnings of analyses. The final result is represented by a black dot with a red border

LCRAnnotationsDB is a database of LCRs identified in the canonical collection of protein sequences from UniProtKB/Swiss-Prot using SEG method with the strict and default parameter sets [16, 22]. Each LCR record in LCRAnnotationsDB includes annotations from publicly available databases (UniProtKB/Swiss-Prot [23], NCBI RefSeq [24], RCSB PDB [25], neXtProt [26], InterPro [5], TOPDOM [27], DisProt [21], ELM [28], PhaSepDB [29], PhaSePro [30]), as well as annotations from predictive methods (IUPred3 [31], Phobius [32]). Annotations related to similar functions are grouped into categories, which we have introduced as part of LCRAnnotationsDB. Where possible, these categories are linked to matching GO terms, which provide hierarchical relationships between them. Note that GO terms related to categories describe the function of a particular LCR fragment they annotate. The category system was inspired by InterPro and DisProt databases, from which we collected some of categories [5, 21]. We also assigned and manually curated our own categories. The data integration is shown in Fig. 1. Additionally, we designed a user-friendly web interface that allows users to easily browse and find information about the biological role of LCRs (https://lcrannotdb.lcr-lab.org/). Users can also automate their analyses and download available data using the RESTful API (https://lcrannotdb.lcr-lab.org/api/).

#### Identification of LCRs

The LCRAnnotationsDB database contains LCRs identified by the SEG method [16, 22]. SEG has three parameters: window size (*W*), trigger complexity ($K_2(1)$), and extension complexity ($K_2(2)$). It uses a sliding protein sequence window of a given size (*W*) to identify a highly biased region. As it scans the sequence, it calculates entropy in the sliding window. If the entropy drops below the $K_2(1)$ threshold, the algorithm starts to expand the identified region until it reaches the $K_2(2)$ threshold. We used two parameter sets of SEG to detect LCRs: strict (*W* = 15, $K_2(1)$ = 1.5, $K_2(2)$ = 1.8) [33] and default (*W* = 12, $K_2(1)$ = 2.2, $K_2(2)$ = 2.5) [16]. The strict parameters detect more regular and shorter LCRs that mostly consist of repetitive patterns [33]. Regular LCRs are composed of homogenous sequences, repetitive fragments, or slightly irregular fragments. Default parameters extend the output of the strict parameters to include more regions that are irregular in composition.

#### Sources of data

As sources of data for LCRAnnotationsDB, we chose publicly available databases and methods for protein region identification that provide information about protein-protein interactions, structures and functional annotations. We selected diverse types of databases, including those that store general data about various types of protein regions and those that specialize in a particular subset (e.g., RCSB PDB, which stores protein structures) [25]. Our final result is a database integrating 12 sources of information. In LCRAnnotationsDB, we integrated annotations from 10 databases (UniProtKB/Swiss-Prot, NCBI RefSeq, RCSB PDB, neXtProt, InterPro, TOPDOM, DisProt, ELM, PhaSepDB, PhaSePro) and 2 predictive methods (IUPred3, Phobius). In Supplementary Material 1, we describe these sources and their methods of use.

The number of annotations derived from each source is presented in Table 1. The largest number of annotations comes from InterPro, neXtProt, and UniProtKB databases, each providing over 1 million annotations. There are also over 200 thousand annotations each from RCSB PDB and TOPDOM. From NCBI RefSeq, DisProt, ELM, PhaSepDB, and PhaSePro, we obtained 27,426; 3,879; 640; 455; and 97 LCR annotations, respectively. We also used two predictors. The first one, Phobius found over 500 thousand annotations of LCRs. The second method, IUPred, identified 200 thousand annotations associated with LCRs.

**Table 1:** The databases used to acquire data for LCRAnnotationsDB. Strict LCRs are fragments identified by SEG with strict parameters and the default LCRs are identified with the default parameters of SEG

Source	Number of strict LCRs annotations	Number of default LCRs annotations
InterPro	18,132	1,465,584
nextProt	88,035	1,193,878
UniProtKB	61,995	1,031,041
Phobius	19,256	542,215
PDB	10,252	290,022
TOPDOM	3,715	266,498
IUpred	16,047	213,487
RefSeq NCBI	547	27,426
DisProt	441	3,879
PhaSepDB	202	640
ELM	23	455
PhaSePro	26	97

#### Categories

Annotations in biological databases lack standardized names, resulting in similar or identical functional annotations being listed under different names across databases. In our database, we store original terms but link them to a category system that groups similar annotations. For instance, in the UniProtKB database disordered regions are annotated as *Disordered*, while the DisProt database annotates them as *disorder*. To unify these nearly identical annotations, we added a category that describes all the available names using a single category named *disordered region*.

Category names and their relationships are based on GO terms. However, in our database we use GO terms to annotate the function of a particular low complexity fragment of a protein sequence, unlike the regular GO database which annotates the whole protein. It may happen that a single annotation retrieved from a source database may point to multiple categories. For instance, LCR at position 608-624 from translation initiation factor IF-2 protein (UniProtACC: A1R516) is annotated in the InterPro database as ‘Translational (tr)-type GTP-binding domain’ (InterPro: IPR000795). This annotation is represented by two categories in our database: ‘GTPase activity’ (GO ID: GO:0003924) and ‘GTP binding’ (GO ID: GO:0005525). Where possible, we have integrated annotation categories with assigned GO from InterPro and DisProt databases. If a given annotation was unique to LCRs and missing from the set of GO terms, we manually created a new category.

The categories assigned manually are mostly based on GO terms. Additionally, some annotations related to structures, mutations, and bond types have no assigned GO terms and are, therefore, not linked to the GO hierarchy. In this database version, we focused on annotations of LCRs identified with strict parameters without any category. The most popular categories were given the highest priority in categorizing annotations. To automatically assign categories to all annotations, we created a set of keywords or sentences in annotations with assigned category names (see Supplementary Material 2 for assignment conditions). The categories were assigned manually through searches of sources, databases, and literature. Additionally, Gene Ontology terms of proteins with annotation were compared, and if any were suitable as a category for the annotations, they were assigned as a category with Gene Ontology terms. If there was a popular annotation without any suitable Gene Ontology term, a new one was created. After the application of categories to all annotations through keywords, their correctness was checked manually. For instance, the annotation ‘alpha-helix’ is categorized as ‘helix’ because there is no GO term describing this specific structural state. The annotation ‘zinc transporter’ is assigned the ‘zinc ion binding’ category with GO ID ‘GO:0008270’ and annotations with the words ‘RNA’ and ‘binding’ are in the ‘RNA binding” category (GO ID ‘GO:0003723’).

#### User interface and implementation

**Fig. 2:**
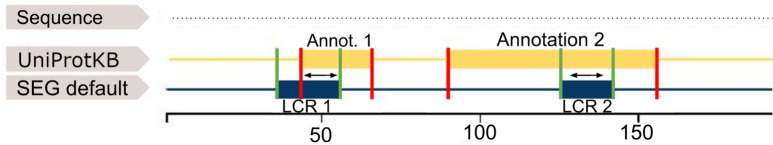
Visualization of annotations and LCR coverage. The double black arrows indicate the coverage of LCRs and annotations. The green lines indicate the boundaries of LCR regions, and the red lines define the boundaries of annotations. Based on regions from Fig. 2, Coverage of LCR by annotation is calculated as 100%*(length-of-coverage/length-of-LCR). Similarly, the coverage of annotation by LCR is 100%*(length-of-coverage/length-of-annotation)

On the Home page, the search form allows users to query proteins, LCRs, or annotations by UniProtACC, protein name, categories, and GO terms. It is also possible to select the source of annotations and to set LCR coverage by annotation, and *vice versa*. Coverage of LCR by annotation is the ratio of overlap length between the annotation region and LCR divided by the total LCR length, while coverage of annotation by LCR is the same overlap with respect to annotation length, expressed in percentages (see Fig. 2).

**Fig. 3:**
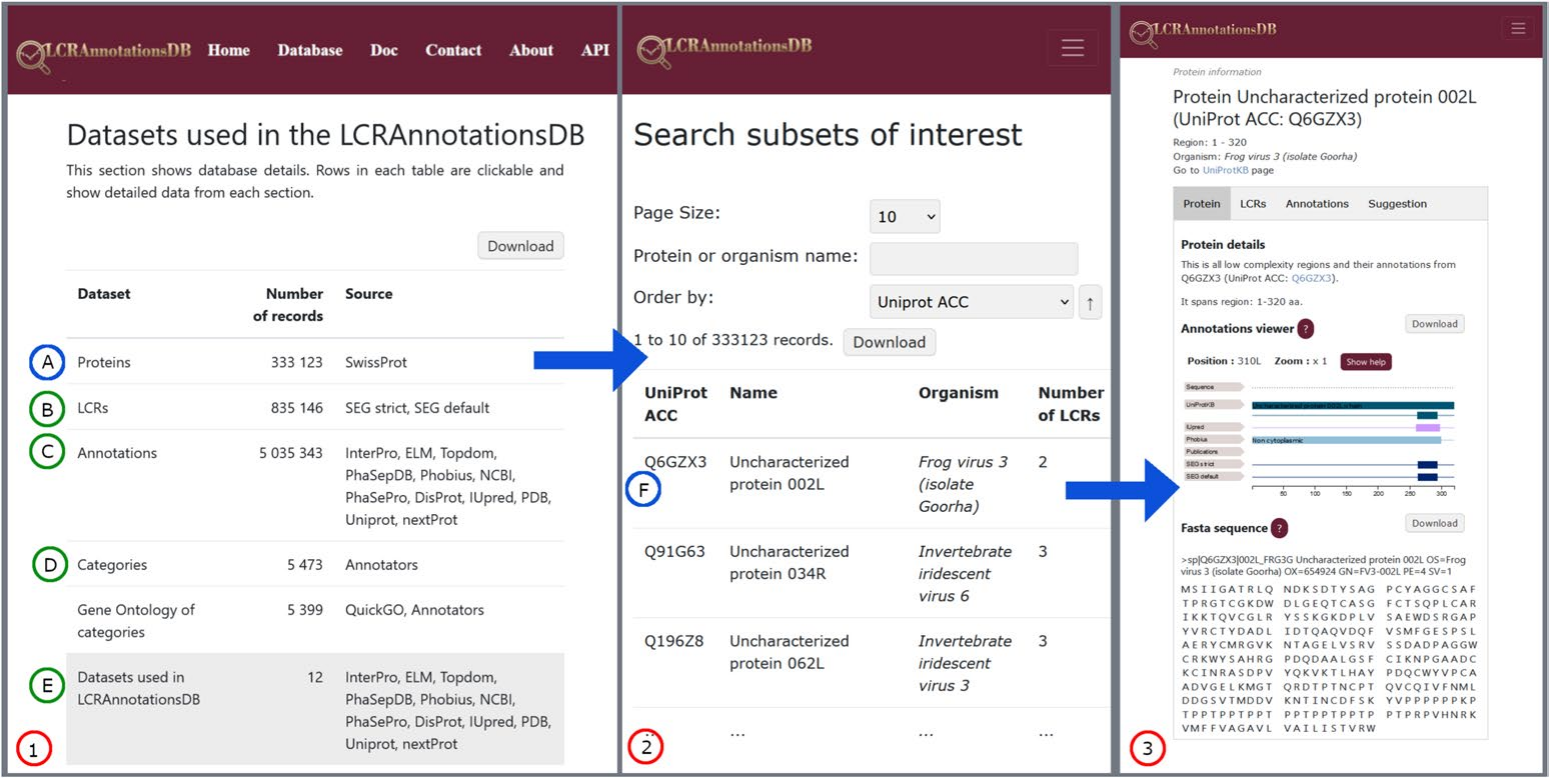
On the Database page (**1**), users can select subpages containing tables of data from our database: Proteins, LCRs, Annotations, Categories, GO and Source Datasets (rows **A**-**E**). For example, clicking on the “Proteins” row (**A**) in Panel (**1**) opens Panel (**2**). Here, clicking on a specific protein, such as “Uncharacterized Protein 002L” with UniProtACC Q6CZX3 (row **F**), will open a Protein subpage in Panel (**3**) with detailed information about this protein

The Database page can be accessed from the top menu, and its main role is to present information stored in the database from different perspectives. Here, users can select the following subpages: Proteins, LCRs, Data Sources, Categories, and GOs (see Fig. 3).

**Fig. 4:**
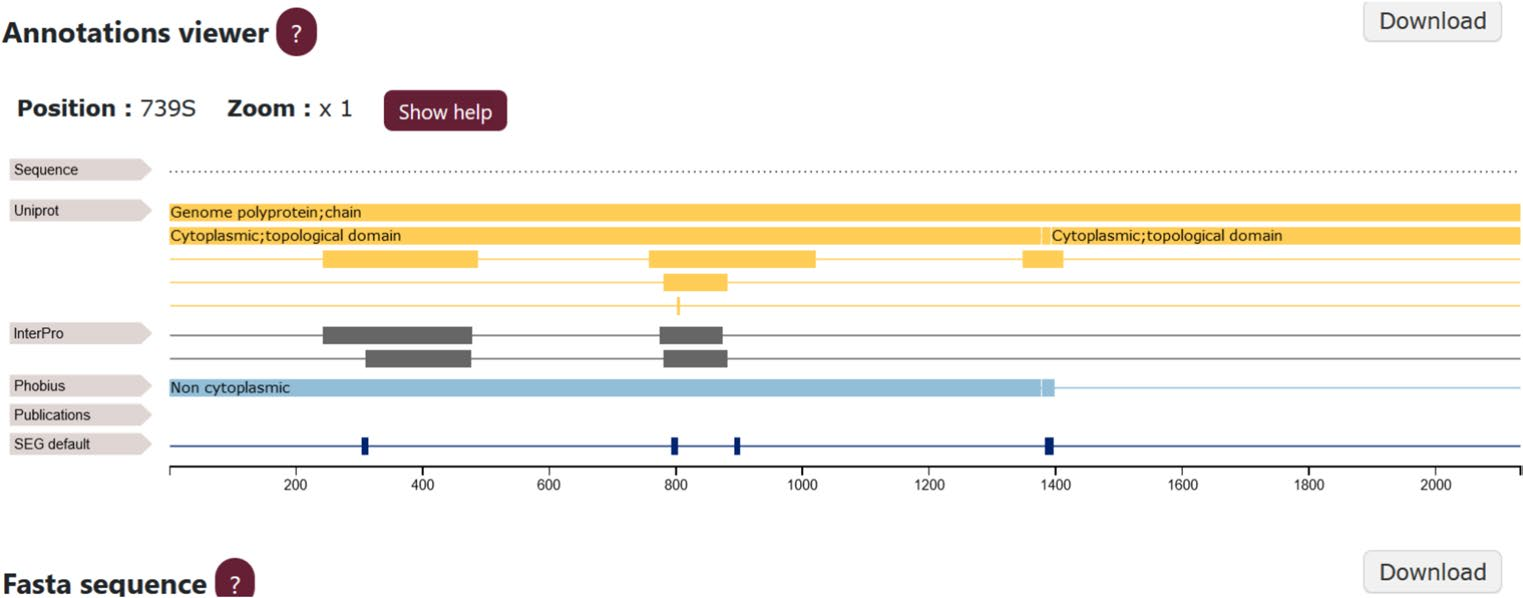
Feature viewer showing the Protein subpage with all LCRs and annotations. An analogous feature viewer is used in the LCR and Annotation subpages. The LCR subpage shows only LCR annotations, whereas the Annotation subpage shows all LCRs that represent a specific annotation

The Protein subpage contains all identified LCRs with their annotations. The LCR subpage includes information about a selected LCR and its annotations. The Annotation subpage presents LCR locations of a selected annotation. The Data Sources table contains a list of annotations and allows users to browse annotations only from a selected source. The Category subpage contains information about proteins, annotations, and LCRs, that have a category assigned. Finally, the GO table contains only categories that have assigned GOs. Each subpage provides a table with a list of corresponding records, which users can browse in detail. After selecting a record, the relevant LCR, protein, or annotation is displayed in a feature viewer on its corresponding subpage. The user can browse regions in a graphical representation and the relationship between LCRs and other annotations. If a region is selected in the feature viewer (see Fig. 4), it is also highlighted in a FASTA sequence displayed below the feature viewer. In each subpage with the graphical representation, the user can download a sequence in FASTA format and view the results presented in the feature viewer as a picture.

**Fig. 5:**
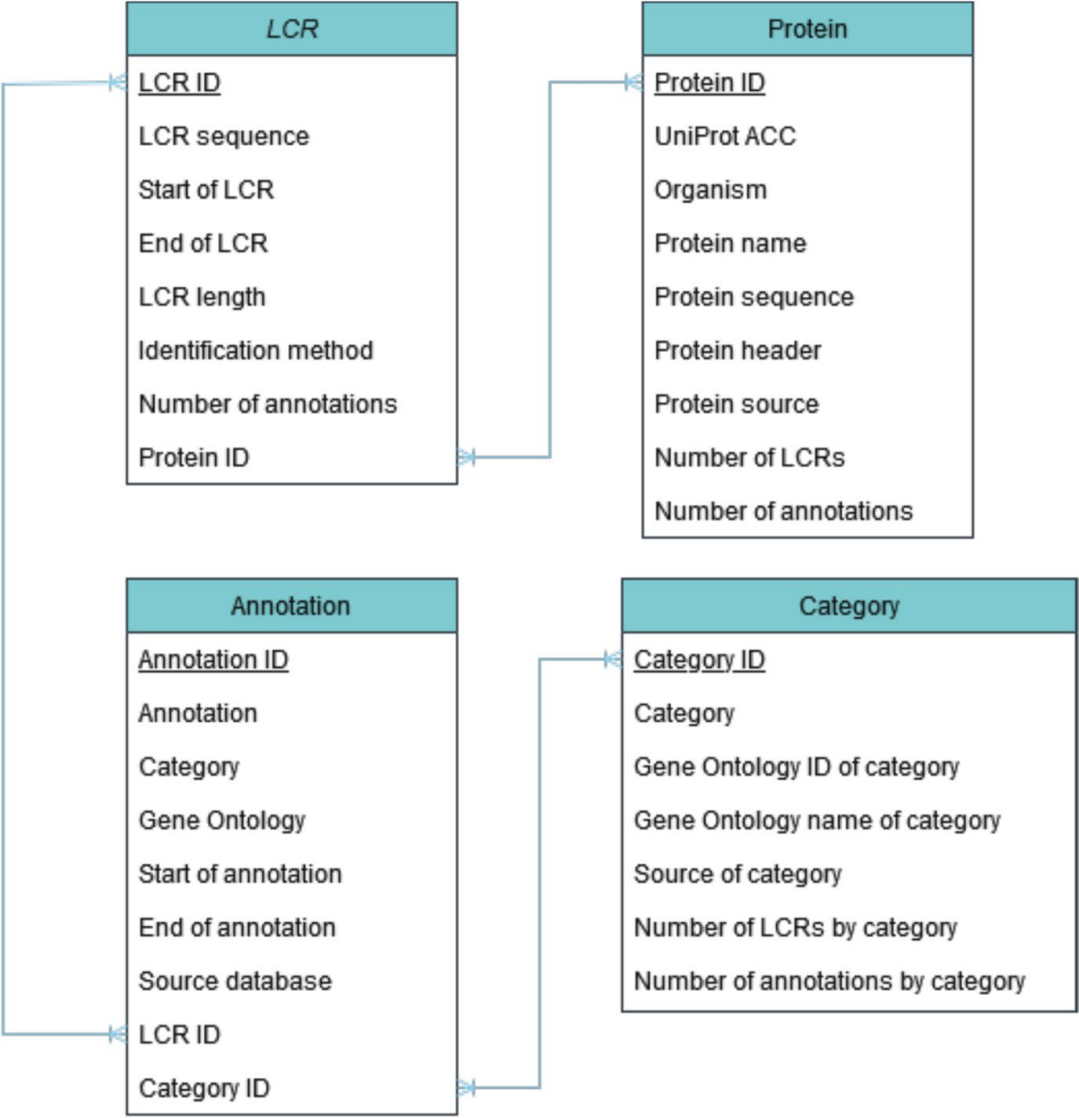
Schema of the PostgreSQL database of LCRAnnotationsDB. The figure shows only the most important columns and tables from the database

LCRAnnotationsDB uses PostgreSQL to store data about proteins and annotations. Additionally, the database stores information about LCRs, proteins, GO, and categories (see Fig. 5). The data integration pipeline is written in Python 3.6., and the web server is supported by the Django framework.

The RESTful API allows users to download data directly from LCRAnnotationsDB in CSV or JSON format (https://lcrannotdb.lcr-lab.org/api/). The API landing page describes the interface details of the RESTful API, which can be used by external software.

### Content

We identified 26,977 LCRs using SEG with strict parameters (which we further call strict LCRs) in 16,798 proteins and 808,169 LCRs using default parameters (default LCRs) in 333,123 proteins out of 563,579 proteins initially in the analysis. The average length of LCRs is 23.9 amino acids for strict and 15.3 amino acids for default parameters. Table 1 presents the number of annotations assigned by all databases and prediction methods. On average, each strict LCR has 9.7 assigned annotations, while a default LCR has 9.4. All of the LCRs in the database have at least one annotation. Since annotations from different databases can provide redundant functional information, we have created categories that group similar functions occurring in various source databases simultaneously. Our database contains 5,479 categories, with each category describing, on average, 886.8 annotations. For each annotation integrated from InterPro, we assigned categories with the same GO term that was assigned in the source database. Analogously, we assigned only those categories from DisProt that had GO term annotations. In summary, we assigned annotations to 5,347 categories based on InterPro, 118 based on DisProt, and manually assigned annotations to 98 categories. One category may come from more than one source. In total, we assigned categories to 66.9% of all annotations for strict LCRs and 63.2 % for default LCRs. We also assigned GO terms to categories where possible (e.g., GO:0016020 term to the “membrane” category). The most popular categories for strict LCRs in the database are as follow: positional variant, disordered region, compositionally biased region, tertiary structure, acidic region, isoform, structural constituent of ribosome, ribosome, translation, translation initiation factor activity, membrane, and ATP binding (see Table 2). Additionally, users can submit suggestions of new annotations and categories supported by evidence from scientific and reliable sources or updates of protein sequences through the suggestion subpage on the web interface.

The LCRAnnotationsDB interface may be used to search, browse, and download data. We provide users with a search form (Home page), annotations (Database), documentation (DOC), contact form (Contact), information about authors (About), and an API manual (API). Additionally, they can use detailed pages describing individual LCRs, proteins, annotations or categories. Detailed pages contain visualizations of sequences with additional information about them. The RESTful API may be used to automate tasks on data stored in the database. For instance, users can download data related to a particular category.

**Table 2:** The 30 most frequent annotations of LCRs identified by the SEG method with strict (strict LCRs) and default (default LCRs) parameter sets, assigned to categories integrated into LCRAnnotationsDB

Category	Number of strict LCRs annotations	Number of default LCRs annotations
Positional variant	79,109	1,072,395
Tertiary structure	14,228	596,004
Membrane	1,652	415,129
Disordered region	20,496	309,241
ATP binding	1,436	201,397
Synthase	429	159,984
Helix	1,231	158,220
Compositionally biased region	14,530	127,434
Translation	2,004	95,429
Structural constituent of ribosome	2,112	91,553
DNA binding	1,375	84,529
Ribosome	2,081	80,732
Cytoplasm	183	66,479
Acidic region	5,998	59,560
RNA binding	1,182	64,068
Signal peptide	1,339	55,359
Nucleoside-triphosphatase regulator activity	267	55,187
GTP binding	462	51,676
Post-translational modification	1,408	49,921
Nucleotide binding	67	50,971
Isoform	2,516	48,287
Translation initiation factor activity	1,830	41,284
Secondary structure	604	40,669
Protein binding	829	35,774
Oxidoreductase activity	57	35,522
Transmembrane transport	78	35,171
Coiled-coil	1,128	29,760
Structure	1,128	29,760
GTPase activity	480	28,923
Magnesium ion binding	114	28,758

In our database, most categories describe the structure of LCRs (tertiary structure, disordered region, helix, secondary structure, coiled-coil, structure, structural constituent of ribosome). There are also annotations of binding regions or nucleotides (RNA, DNA, ATP, GTP binding), ions and proteins. Additionally, in LCRAnnotationsDB, some categories refer to participation in biological processes (e.g., GTPase activity, translation initiation factor activity). There are also numerous positional variants of human proteins from the neXtProt database and more general categories like ‘repeat’.

### Enrichment of LCR annotations in protein families

LCRs are short fragments of the complete protein sequence. To investigate the relationship between functional annotations of LCRs and global protein functions, we conducted an analysis of the enrichment of LCR annotations in InterPro Families set (InterPro version 99.0). First, we retrieved a dataset from our database that consisted of proteins with annotated LCRs, which overlap by at least 70% with the annotation and vice versa (the LCR dataset). This means that the LCR covers the annotation in at least 70% of the annotation length and that the annotation covers the LCR in at least 70% of the LCR length. We assume that in such cases the annotations are directly related to the presence of LCRs. Then, for each protein sequence from the LCR dataset, we retrieved the IDs of InterPro Families assigned to it. Having a list of InterPro IDs, we then downloaded all proteins from InterPro belonging to those families. We call this the InterPro dataset. In this analysis, we excluded annotations with InterPro Family IDs from the LCR dataset. This ensures that the analyzed Protein Family IDs are related to the entire protein sequence, allowing us to hypothesize about a possible relationship between the existence of LCRs and protein function. According to the InterPro Families definition, the annotation of a protein represents its whole length (https://interpro-documentation.readthedocs.io/en/latest/entries_info.html). For both the LCR and InterPro datasets, we then counted the number of sequences in each dataset separately and the number of sequences that belong to the intersection of both sets. Finally, to analyze the enrichment of LCR annotations in protein families, we calculated the hypergeometric tests:

1 $$\begin{aligned} P(X)=\frac{{\left( \begin{array}{c}k\\ x\end{array}\right) }{\left( \begin{array}{c}N-k\\ n-x\end{array}\right) }}{{\left( \begin{array}{c}N\\ n\end{array}\right) }}. \end{aligned}$$

where *N* denotes the number of proteins in UniProtKB/Swiss-Prot, *n* is the number of proteins in the LCR dataset, *k* is the number of proteins in the InterPro dataset, and *x* is the number of proteins from the intersection of both sets. The resulting p-values were adjusted using the Benjamini-Hochberg multiple-testing correction procedure, assuming a false discovery rate of 5%.

We found that more than half (56.54%) of the LCR annotations in our database are significantly enriched within InterPro Families set. These results may indicate a strong relationship between the presence of an LCR and the general protein function in the dataset analysed. To analyze the results in aggregated form, we grouped the annotations by their categories. We observed many significant annotations related to structural sequence features such as helix, secondary and tertiary structure, and coiled-coil. Other important categories included RNA binding, TAR and RRE motifs, and the RGG box. The aggregated results for the most frequent categories are presented in Table 3. All results of the enrichment analysis are available in Supplementary Material 3.

**Table 3:** Number of significant results from the hypergeometric test with Benjamini-Hochberg procedure. The most frequent family categories in this table are structural and nucleotide binding terms

Category name	Number of significant tests	Percentage of significant tests
Membrane	6,711	70.36%
Compositionally biased region	4,222	45.93%
Helix	3,972	58.27%
Acidic region	2,768	59.49%
Signal peptide	2,385	53.42%
Disordered region	1,944	29.64%
Secondary structure	1,575	47.67%
Isoform	496	92.02%
Tertiary structure	458	100.0%
Structure	443	80.55%
Coiled-coil	443	80.55%
Repeats	416	100.0%
Positional variant	269	100.0%
Nuclear import signal receptor activity	164	95.91%
Sequence conflict	127	100.0%
DNA binding	120	100.0%
Protein binding	116	100.0%
ATP binding	62	95.38%
Zinc finger	41	100.0%
RNA binding	37	100.0%
GTP binding	33	94.29%
Translation	28	100.0%
ATP hydrolysis activity	28	90.32%
Zinc ion binding	28	100.0%
Ribosome	26	100.0%
Structural constituent of ribosome	26	100.0%
Regulation of DNA-templated transcription	26	100.0%
Sequence variant	25	100.0%
Synthase	24	100.0%
DNA-binding transcription factor activity	23	100.0%
All	27,615	56.54%

## Utility and discussion

### Use case

#### LCRs with annotations in protein families

Previous sections of the paper focused on the usability of our database and a description of its content. In this part of the paper, we provide an in-depth examination of specific cases of categorized annotations that exhibit significant enrichment within a family. We describe these families and compare the sequences of LCRs to determine whether these regions may share similar annotations. Additionally, we identify proteins with unannotated LCRs and speculate on their possible functions.

To demonstrate the association between LCRs and protein families, we executed a series of steps. Initially, we obtained families significantly enriched in LCR annotations, as described in the previous section. To identify the most interesting pairs, we determined the proportion of proteins with annotated LCRs within each protein family. We filtered out (1) pairs with a frequency of less than 30% in datasets of InterPro protein families, (2) pairs with a frequency of less than 30% in datasets of proteins with annotated LCRs, and (3) pairs with fewer than 5 common proteins between datasets. Additionally, we compared GO of (1) proteins from a particular family, (2) categories of its LCR annotations and (3) GOs of families. GOs come from (1) the QuickGo database, (2) GOs of categories sourced from LCRAnnotationsDB, and (3) information about families from the InterPro database.

**Fig. 6:**
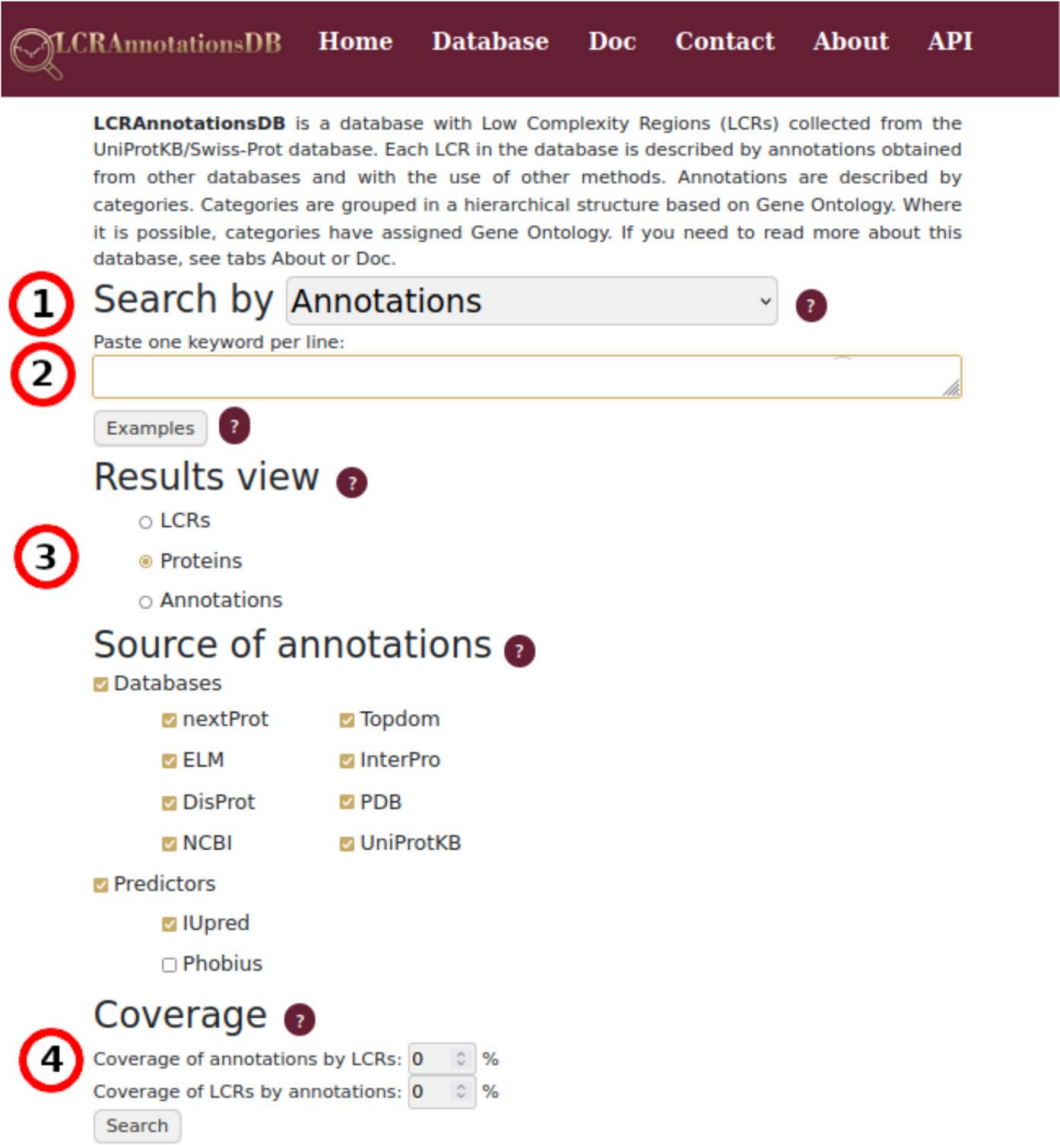
Main form for querying LCRAnnotationsDB. The form allows searching for proteins, LCRs, and annotations using different criteria: the user can (**1**) select the type of query, (**2**) enter the keywords for the query, (**3**) select the result view, and (**4**) set the minimum LCR coverage and annotation coverage to filter the results. To search for protein families from InterPro, select the type of query as annotations (**1**), select protein result view (**3**), set the coverage to 70% (**4**), and enter the family name in the keywords (**2**). To find annotations from the analysis, select the type of query as annotation (**1**), select annotations result view (**3**), set the coverage to 70% (**4**), and enter the annotation name in the keywords (**2**). It is also possible to search annotations using UniProtACC by changing (**1**) to UniProtACC

Consequently, we obtained 49 categories of LCR annotations relevant to the categories of LCR datasets with unique pairs of 506 InterPro and LCR datasets. A substantial fraction of these categories (around 33%) are linked to nucleotide binding or structural states, underscoring the significance of these regions in molecular pathways and protein structure determination. Notably, our focus was drawn to the category of RNA binding proteins, including three distinct families: the anti-repression trans-activator protein (REV) family (InterPro Entry: IPR000625), the immunodeficiency virus transactivating regulatory protein (Tat) family (IPR001831), and the RNA-dependent RNA polymerase (RdRp) family (IPR002166). These families are involved in various facets of viral RNA metabolism, including transcription, translation, and replication. To identify the proteins from InterPro families with annotated LCRs described in this chapter, users may conduct a search using the form view illustrated in Fig. 6.

Firstly, we analyzed the anti-repression trans-activator protein (REV) family (InterPro Entry: IPR000625), which comprises proteins from immunodeficiency viruses essential for viral propagation. REV proteins possess an RRE-binding motif, enabling them to bind the Rev response element (RRE) to form complexes with mRNA. The arginine-rich RRE-binding motif serves as a nuclear localization signal, binding RRE-containing RNAs to form a complex for nuclear export [8, 9]. REV proteins involved in the export pathway of viral mRNA, form a complex with REV protein dimers, mRNA, and host proteins such as CRM1 to facilitate the translocation of viral RNA from the nucleus to the cytoplasm [9, 34]. The objective is to shield viral mRNA from detection as foreign. The InterPro database lists 74 proteins from the UniProtKB/Swiss-Prot database in this family were analyzed. Utilizing LCRAnnotationsDB, we identified 71 proteins belonging to the REV family with LCRs overlapping with the RRE-binding motif annotations. Furthermore, we found 73 proteins with RRE motif annotations, all corresponding to UniProtKB REV proteins. Among these proteins, 71 belonged to the InterPro REV family. Interestingly, two of them (UniProtACC: P36339, P22379) were not categorized in InterPro Families as REV proteins, even though their sequences show 70% LCR coverage in the RRE motif. This suggests the need for further investigation for the potential classification of these proteins within this family.

We noticed that some GOs of proteins from this family overlap with categories of LCR annotations. Three of GOs (host cell nucleus, regulation of DNA-templated transcription, DNA-binding transcription factor activity) are assigned to the REV family and categories of LCR annotations. The other three GOs (RNA binding, protein export from nucleus, protein localization to nucleoplasm) describe only categories and proteins. There is only one GO annotation of a protein, nuclear import signal receptor activity, that is not assigned to LCR annotations. Additionally, more precise GO categories, such as mRNA binding, host cell cytoplasm, and host cell nucleolus, are assigned to categories of LCR annotations. Some proteins are categorized under the viral process.

Secondly, we analyzed the immunodeficiency virus transactivating regulatory protein family, Tat (IPR001831). These proteins work with polymerase and the transcription elongation complex in viral RNA synthesis [35]. Specifically, Tat recruits the transcription elongation factor pTEFb, the TATA box binding protein, and chromatin-modifying proteins to the LTR promoter, leading to RNA polymerase II phosphorylation, the assembly of novel transcription complexes, and chromatin remodeling [36–39]. Proteins in the Tat family contain the TAR-binding motif, which binds the trans-activating response element [40]. The TAR-binding motif in Tat is a critical functional component of this protein family, as it binds the RNA stem-loop structure in viral RNAs [35, 41]. Some studies suggest that Tat can also enhance viral transcription independently of binding the trans-activating response element (TAR) located in the viral transcripts [42, 43]. Tat’s involvement extends to HIV-1 RNA splicing, capping, translation, and reverse transcription [44–47]. InterPro indicates that this family contains 72 proteins from the UniProtKB/Swiss-Prot database, originating from human, simian, and bovine immunodeficiency viruses, as well as equine infectious anemia and Jembrana disease viruses. Using LCRAnnotationsDB, we identified 23 proteins with LCRs in the TAR-binding region, all meeting the 70% coverage threshold. Additionally, nine proteins showed TAR-binding motif LCRs below 70% coverage. The remaining 40 proteins had TAR-binding motif annotations in the UniProtKB/Swiss-Prot database, but these motifs were not classified as LCRs, due to greater diversity compared to the strict and default SEG thresholds.

For this family, we also compared the GOs of proteins and categories of LCRs. Only one GO - nuclear import signal receptor activity - was assigned to over 50% of the proteins and was not assigned to any other category. The GOs of families (positive regulation of viral transcription, RNA-binding transcription regulator activity, host cell nucleus) were not assigned to 9 proteins from this family and were not assigned to categories of LCR annotations for only one protein from this family. Categories for at least half of the proteins had additional GOs assigned, including protein serine/threonine phosphatase inhibitor activity, virus-mediated perturbation of host defense response, symbiont-mediated suppression of host type I interferon-mediated signaling pathway, metal ion binding, actinin binding, extracellular region, negative regulation of peptidyl-threonine phosphorylation, symbiont-mediated suppression of host translation initiation, host cell cytoplasm, apoptotic process, DNA-templated transcription, and cyclin binding.

The final group analyzed was the RNA-dependent RNA polymerase (RdRp) family (IPR002166), consisting of 56 proteins from the UniProtKB/Swiss-Prot database. This family includes viral ‘RNA-directed RNA polymerases’ and ‘genome polyprotein proteins’, which are involved in RNA replication [48, 49]. In LCRAnnotationsDB, 53 out of 57 proteins in this family contain at least one LCR. Among them, 18 proteins have LCRs in the RNA-binding motif, each surpassing 70% coverage. Another 11 proteins, classified as genome polyprotein proteins, showed lower coverage for LCR and RNA-binding annotations. The remaining 19 RNA-directed RNA polymerase proteins and 5 genome polyproteins lacked RNA-binding annotations in any source database. We hypothesize that all LCRs from this family, located in approximately the same position and rich in arginine, can be considered as RNA-binding regions.

In the RdRp family, there were four GOs assigned to proteins that were not assigned to annotations: protein binding, viral process, nuclear import signal receptor activity, and virion component. We also found 36 GOs assigned to categories but not to proteins. The most common ones were RNA-templated transcription, 5’-3’ RNA polymerase activity, and virus-mediated perturbation of host defense response. At least one category of LCR annotations was assigned to 57 proteins outside the family. Over 20 proteins from this family did not have GOs assigned.

The results of each of these three family analyses illustrate diverse issues related to database usage. The study of the REV family revealed proteins not included in the family. LCRAnnotationsDB allowed us to find and include them. The Tat family, on the other hand, contains an irregular LCR annotated as the TAR-binding motif. In some Tat family proteins, this region was not identified as an LCR. This suggests that TAR-binding LCRs are in the twilight zone of detection, possible due to strong evolutionary pressure on this region. Our database showed the diversity in protein functional region composition within specific families. The issue with the RdRp family was its incomplete annotation in UniProtKB. Some members of the family have annotated LCRs, while others do not. These unannotated proteins contain LCRs rich in arginine with amino acid compositions similar to those with LCR annotations. In all families, proteins and categories of LCRs had more GOs assigned than InterPro Families. Additionally, not all GOs were assigned to each protein within the families. In all analyzed families, more GOs were assigned to LCRs than to protein and family categories. Full data from this analysis can be found in Supplementary Material 4.

#### RNA-binding LCR in prokaryotic large ribosomal subunit protein bL20c

**Fig. 7:**
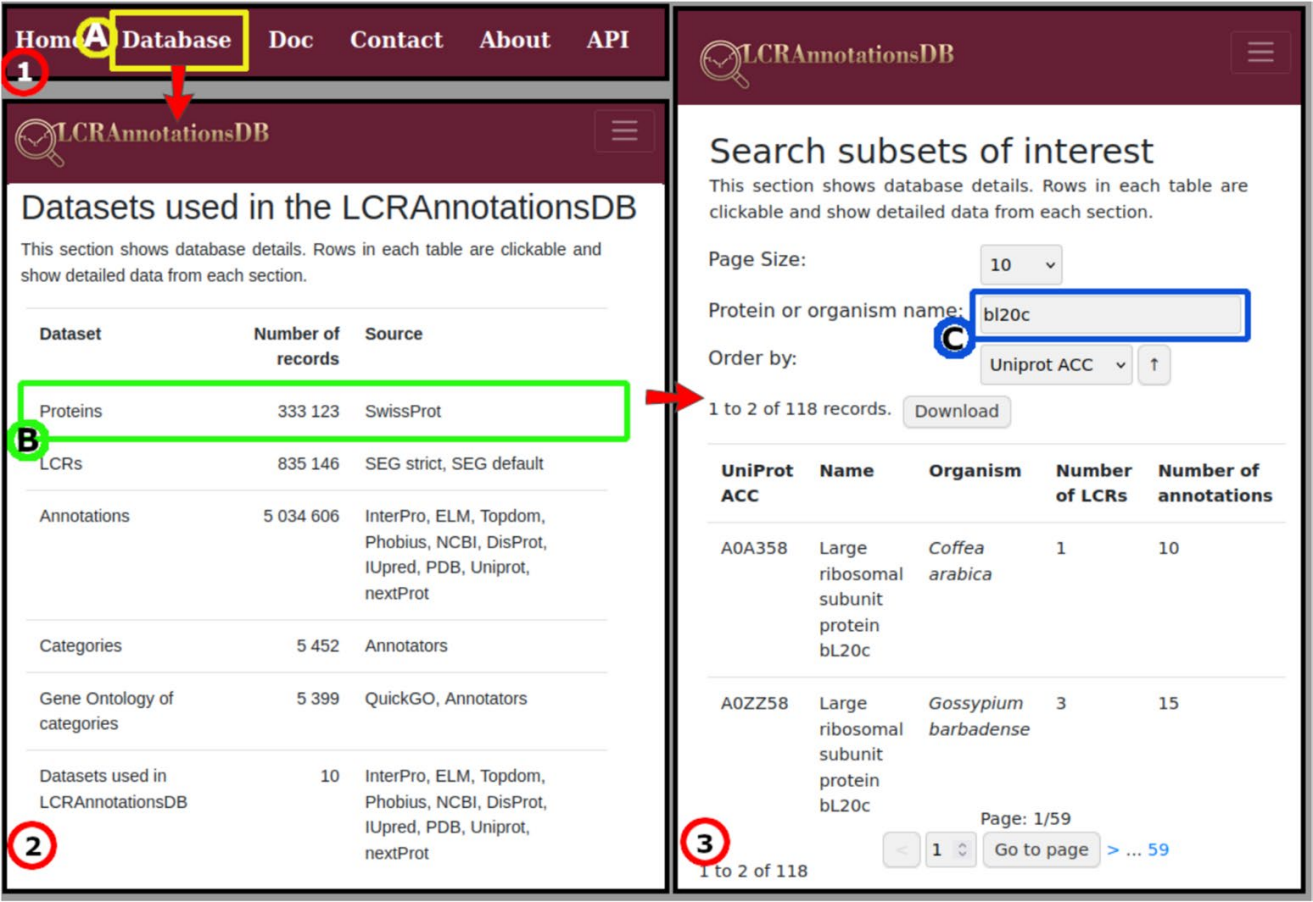
Step-by-step example of searching for bl20c proteins containing LCRs. Select “Database” (in box **A**) view from Panel **(1)**. Then Panel **(2)** shows the datasets that can be browsed. When selecting the “Proteins” dataset (in box **B**), the user is directed to Panel **(3)**, which lists all proteins. Type “bl20c” in the “Protein or organism name” field (in box **C**) and browse all bl20c proteins that contain at least one LCR

Out of many proteins with RNA-binding motifs in LCRAnnotationsDB, we analyzed those containing more than one annotated LCR. We investigated this set manually and found that a subset of proteins annotated as both RNA- and protein-binding is the most interesting. This means these proteins possess both RNA-binding and protein-binding LCRs. We excluded the most obvious examples, such as serine/arginine repetitive matrix protein 2 (Q9UQ35), which has a known structure and mechanism of action. The most intriguing was the prokaryotic 50S subunit bL20, a protein with a vaguely known function [50, 51]. Using the LCRAnnotationsDB search by protein name, we identified 118 homologous proteins (see description of the search process in Fig. 7). The SEG method with strict parameters is not always able to identify both LCRs in each case, but the protein can bind both types of molecules. Several very recent articles show how bL20 participates in ribosome assembly [52–54]. bL20 is a crucial piece in the ribosome assembly, forming the L20 block, which binds the assembly core at the early stages of the process. The element of interest is the RNA-binding motif, rich in arginines, located at the N-terminal of the protein. The C terminus has a protein-binding motif rich in lysines, which is typical for this role. Arginine stretches are known to bind RNA [6, 7], and in this case, they specifically bind rRNA [52]. The protein-binding motif binds to elements of the Core and the L20 block [52].

### Discussion

In the digital space, a plethora of general and more specific databases exist, offering annotations for protein sequences. These resources predominantly focus on high-complexity regions, such as protein domains, families, entire proteins, and complexes, which may include a subset of annotations pertaining to LCRs. Despite this, a dedicated database that exclusively focuses on the functions and structures of LCRs is absent. This deficiency makes retrieving LCR-specific annotations difficult and time-consuming. As a result, researchers frequently default to applying GO terms for the functional analysis of LCRs. While this provides a broad categorization for proteins containing LCRs, it falls short of delineating the specific functions of the LCRs themselves. The demand for such a specialized database has been consistently expressed by researchers engaged in exploring the Dark Proteome [55].

Annotations in biological databases are assigned in two ways: manually or automatically, with the support of different tools such as domain predictors or text mining methods [56, 57]. One challenge that arises when creating meta-databases is unifying data that are redundant across databases using different labels to describe similar biological phenomena. To address this issue, we designed a category system in which the same annotations, named differently in distinct data sources, are represented by a single category. Grouping annotations into categories allows us to analyze the functions of LCRs on a more general level, providing novel insights into LCRs and their relation to structural sequence features or protein functions. Most categories have assigned GO terms; however, some structural and other categories, such as coiled coils, secondary structures, or isoforms, lack GO term assignments. Similar systems are used in other databases, such as DisProt and InterPro. However, we must account for the strong bias of the UniProtKB/Swiss-Prot database in our analysis. This bias may result from the small size of the database and limited knowledge about specific organisms, processes, and proteins. Additionally, we integrated some biased and highly specialized databases, like DisProt or neXtProt, which focused on narrow groups of proteins.

The significant enrichment of LCR annotations in some InterPro protein families shows that low complexity fragments in proteins can be strongly related to general protein function. These relationships occur between annotations from very diverse categories and families and can be observed for more than 50% of the annotations in our dataset.

The analysis of the co-occurrence of InterPro Families and LCR annotations shows that we can distinguish many functional LCR groups. This type of analysis may serve as an alternative to predicting protein region function based on similarity. We selected three example families: REV, TAR, and RdRP. Proteins belonging to different families play different roles in organisms, although all contain similar RNA-binding regions. This LCR group, common to the three different protein families, is known as the arginine-rich RNA-binding domain [6, 7]. We have noticed significant differences between GOs of protein families, proteins, and categories of LCRs. We suspect that analyzing GOs solely of proteins enriched with LCRs may overlook some important relationships between the occurrence of LCRs in proteins and their functions. In this case study, we have shown that LCRAnnotationsDB can be used to analyze LCRs in the context of protein families.

The final example of ribosomal subunit protein bL20c analyzes a protein with two LCRs, each possessing a distinct function. This protein features regions that bind to both proteins and DNA, each characterized by unique amino acid compositions. When analyzing these regions using protein GO terms, the LCRs are erroneously classified as having both protein-binding and DNA-binding functions, although each function is realized independently. This analysis highlights the dangers of using general GO terms assigned to proteins to describe their regions. Such descriptions impede a precise annotation of LCRs.

In the future version, we plan to manually add information about LCRs from scientific publications. This approach is used in DisProt where data about intrinsically disordered regions is manually curated based on experimental findings and scientific literature [21]. Similarly, databases such as PhaSePro and PhaSepDB also rely on manual annotation. We have noted that these resources often feature unique data not present in general databases such as UniProtKB or InterPro, highlighting the value of manual annotations in enriching database content. To add general GO terms assigned to categories of LCR annotations, our database invites users to share their research findings (https://lcrannotdb.lcr-lab.org/contact/). We believe that integrating the existing information on LCR functions in one place will enable our tool to facilitate future research on the function of LCRs. Furthermore, incorporating data from author publications significantly enhances the ability to present their results. We also plan to continuously expand our categorization system since not all annotations are currently covered by categories.

These plans are not, however, devoid of challenges. The main challenge is the lack of a precise definition of LCRs [58]. This poses a significant issue for studies aiming to cover the whole LCR space. The subject is complicated by technical issues, such as database redundancy when retrieving data from other sources, the absence of universal standards for data formats, the need for format changes, and annotation bias. These challenges may be addressed through experience and experiments to optimize the method. The final challenge, manual annotation, cannot be resolved by technical means at present. It requires a group of annotators and a working methodology to retrieve data in an organized manner. This kind of endeavor requires a lot of resources and planning.

## Conclusion

We have created LCRAnnotationsDB, a curated and comprehensive database for protein LCR annotations. It consolidates data from various sources and provides a platform to explore the diversity and importance of LCRs in different protein families and functional categories. Additionally, we have systematized annotations into a unified categorical framework to resolve the duplicative nature of annotations found in various databases. Most of these categories correspond to established GO terms; however, some annotations are classified under newly created categories.

We have presented the analysis of LCR sequences within protein families and demonstrated a pronounced enrichment of protein families with annotated LCRs. Our database encourages researchers to easily screen LCRs within the protein sequences of their interest. LCRAnnotationsDB features a user-friendly web interface, complete with a tutorial on its documentation subpage. It can be accessed at https://lcrannotdb.lcr-lab.org/.

Our work fills a gap in the study of protein sequences. Most previous research focused on high-complexity sequences, leaving LCRs heavily understudied.

## Supplementary information

Supplementary Material 1.

Supplementary Material 2.

Supplementary Material 3.

Supplementary Material 4.

## Data Availability

Code to our enrichment analyses are available in repository https://github.com/Addreoran/lcrannotationsdb_enrichment. All data included in LCRAnnotationsDB are available at https://lcrannotdb.lcr-lab.org/.
