# Supplementary material for: LCRAnnotationsDB: a database of low complexity regions functional and structural annotations
Source: BMC Genomics. 2024 Dec 27;25:1251. doi: 10.1186/s12864-024-10960-5 (PMC11674544; doi:10.1186/s12864-024-10960-5)
Supplement: Supplementary file 2 — Supplementary Material 2. [file 12864_2024_10960_MOESM2_ESM.pdf]

1 Supplementary document with a description of data sources for LCRAnnotationsDB

LCRAnnotationsDB collects data about low complexity regions from 12 source databases and predictors. In this supplementary file, we describe these sources and the methods of their integration into LCRAnnotationsDB.

The UniProt Knowledgebase integrates and standardizes data from different sources about protein functions, structures, mutations, bond types, locations, and domains [1]. It contains sequences with automatically annotated and manually curated records. UniProtKB/Swiss-Prot is part of the UniProt Knowledgebase and contains high-quality, manually curated records only. Therefore, it is one of the databases we use to annotate LCRs. We downloaded data from UniProtKB in XML format, which we used for annotations, and in FASTA format, which we used as a source of protein sequences.

NCBI RefSeq is a database with partially curated, non-redundant genome, transcript, and protein databases. NCBI RefSeq contains sequences and annotations from different sources [2]. Data are generated by different pipelines depending on the class of sequences and organisms. For example, archaea and bacteria are annotated using NCBI's prokaryotic genome annotation pipeline. Data in NCBI RefSeq are stored in the GenPept format, which may be accessible through FTP. However, we obtained annotations through the Entrez API (E-utilities) from NCBI RefSeq proteins since it is the most convenient way.

PDB (Protein Data Bank) is a database containing manually curated protein structures [3]. Data come from experiments such as X-ray crystal structure determination, NMR, cryo-electron microscopy, and theoretical modeling from the AlphaFoldDB [4], and RoseTTAFold [5, 6] tools. PDB is integrated with over 40 resources, for example, UniProtKB and NCBI RefSeq, which allows searching protein structures by UniProtACC. Data can be accessed via an FTP server and REST API in many different formats. We collected information on experimental methods from PDB using the API.

neXtProt is a knowledge base about human proteins [7]. As a source of protein sequences, it uses UniProtKB/Swiss-Prot. neXtProt enriches proteins with information about expression, protein-protein binary interactions, functions, post-translational information, variant information, etc. It integrates high quality data with an error rate of information below 5%. The error rate is documented for each record in the metadata and depends on the type of analysis. Records with an error rate between 1% and 5% are marked as silver data [8]. We downloaded neXtProt from neXtProt's FTP as XML files, which represent different chromosomes.

InterPro database integrates the classification of proteins into families and predicts the presence of domains and conserved sites [9]. New versions of InterPro are released every two months. The entire database is regularly checked for accuracy and all new entries are manually curated. InterPro integrates signatures from the following databases: Pfam, HAMAP, MobiDB Lite, PRINTS, Prosite, SFLD, SMART, CATH, CDD, Panther, PIRSF, and SUPERFAMILY [9]. The advantage of InterPro is that it combines the protein signatures from these databases into a single searchable resource, creating a large integrated resource. Annotations from InterPro have GO terms provided for most entries [9]. InterPro allows users to download information about domains from FTP server in TSV format. Data can also be downloaded through the InterPro API.

TOPDOM is a database that contains a collection of domains and motifs located in transmembrane regions of proteins [10]. As input, it uses protein sequences from the UniProtKB database. Then, the CCTOP algorithm selects sequences that discriminate between transmembrane proteins and non-transmembrane proteins. Information from this database can be used in constrained topology prediction methods. An example is a Bayesian type that determines the location of a certain part of the sequence. The process of database creation is automated, so it may be updated on demand. The entries are stored in XML format and may be downloaded from the database website. We have integrated data from TOPDOM version 3.0.

DisProt is a database that contains manually curated Intrinsically Disordered Proteins (IDPs) [11]. IDPs are regions that alone do not create a three-dimensional structure under physiological conditions. However, these regions may still fold while binding other proteins and ligands. Their functions are described by Gene Ontology (GO) or Intrinsically Disordered Protein Ontology (IDPO) [11]. Annotations are provided by the community and reviewed by biocurators. We downloaded data from DisProt as a CSV file. We have integrated data from DisProt version 2023_12.

ELM (Eukaryotic Linear Motif) is a resource of short linear motifs (SLiMs) [12]. These are short motifs that participate in protein-protein binding and are post-translationally modified. Motifs in ELM are assigned to six classes: cleavage, degradation, docking, ligand, modification, and targeting motifs. Records in ELM are highly curated with data from scientific publications. We downloaded data from ELM as a CSV file using the API of this database. We have integrated data from ELM version 1.4.

PhaSepDB provides a collection of manually curated Liquid-Liquid Phase Separation (LLPS) proteins and membraneless organelles related proteins [13]. Each Phase Separation (PS) entry represents PS validation experiments of a protein illustrated in one publication. Each record includes detailed annotations, such as the material states of the PS droplets, PS verification experiments, regions used in experiments, phase diagrams, PS partners, and regulation information. The database is available as a web application or as a set of Excel and CSV files. We used the latter form, which we then processed to extract information about the regions and domains important for proteins to undergo PS. We have integrated data from PhaSepDB version 2.1.

PhaSePro is another database of proteins driving LLPS in living cells [14]. This dataset is manually curated and consists only of experimentally verified cases of LLPS

supported by literature evidence. Each entry in the database provides information on sequence boundaries and characteristics of the experimentally validated LLPS driver region(s). Additionally, entries are enriched with details such as the functional relevance of the LLPS system, types of molecular interactions involved, regulatory mechanisms of LLPS, etc. Access to the database is available via the online interface or through the RESTful API. Users also have the option to download data in JSON, XML, or TSV format. We have integrated data from PhaSePro version 1.1.

Phobius is a predictor that identifies protein topology and signal peptides [15]. Predicted regions are described by the hidden Markov model (HMMs). HMMs reduce errors caused by cross-prediction between regions of interest. We used the binary version, which can be downloaded from the official webpage.

IUPred3 is a method that predicts disordered regions in proteins [16]. This method estimates protein energy to predict protein perturbation. It also uses the biophysical properties of ordered and unordered regions. IUPred3 calculates the occurrence probability of residues in disordered regions. The service is accessible through the web page and RESTful API. We decided to use the API to obtain disordered regions for our database.

References

[1] Uniprot: the universal protein knowledgebase in 2023. Nucleic acids research **51**(D1), 523–531 (2023)

[2] O'Leary, N.A., Wright, M.W., Brister, J.R., Ciufo, S., Haddad, D., McVeigh, R., Rajput, B., Robbertse, B., Smith-White, B., Ako-Adjei, D., Astashyn, A., Badretdin, A., Bào, Y., Blinkova, O., Brover, V., Chetvernin, V., Choi, J., Cox, E., Ermolaeva, O.D., Farrell, C.M., Goldfarb, T., Gupta, T., Haft, D.H., Hatcher, E.L., Hlavina, W., Joardar, V.S., Kodali, V.K., Li, W., Maglott, D.R., Masterson, P., McGarvey, K.M., Murphy, M.R., O'Neill, K., Pujar, S., Rangwala, S.H., Rausch, D., Riddick, L.D., Schoch, C.L., Shkeda, A., Storz, S.S., Sun, H., Thibaud-Nissen, F., Tolstoy, I., Tully, R.E., Vatsan, A.R., Wallin, C., Webb, D., Wu, W., Landrum, M.J., Kimchi, A., Tatusova, T.A., DiCuccio, M., Kitts, P.A., Murphy, T.D., Pruitt, K.D.: Reference sequence (refseq) database at ncbi: current status, taxonomic expansion, and functional annotation. Nucleic Acids Research **44**, 733–745 (2015)

[3] Burley, S.K., Bhikadiya, C., Bi, C., Bittrich, S., Chen, L., Crichlow, G.V., Christie, C.H., Dalenberg, K., Costanzo, L.D., Duarte, J.M., Dutta, S., Feng, Z., Ganesan, S.J., Goodsell, D.S., Ghosh, S., Green, R.K., Guranovic, V., Guzenko, D., Hudson, B.P., Lawson, C.L., Liang, Y., Lowe, R., Namkoong, H., Peisach, E., Persikova, I., Randle, C., Rose, A.S., Rose, Y., Sali, A., Segura, J., Sekharan, M., Shao, C., Tao, Y.-P., Voigt, M., Westbrook, J.D., Young, J.Y., Zardecki, C., Zhuravleva, M.: Rcsb protein data bank: powerful new tools for exploring 3d structures of biological macromolecules for basic and applied research and education in fundamental biology, biomedicine, biotechnology, bioengineering and energy sciences. Nucleic Acids Research **49**, 437–451 (2020)

[4] Váradi, M., Anyango, S., Deshpande, M.S., Nair, S., Natassia, C., Yordanova, G., Yuan, D.Y., Stroe, O., Wood, G., Laydon, A., Zídek, A., Green, T., Tunyasuvunakool, K., Petersen, S., Jumper, J.M., Clancy, E., Green, R., Vora, A., Lutfi, M., Figurnov, M., Cowie, A., Hobbs, N., Kohli, P., Kleywegt, G.J., Birney, E., Hassabis, D., Velankar, S.: Alphafold protein structure database: massively expanding the structural coverage of protein-sequence space with high-accuracy models. Nucleic Acids Research **50**, 439–444 (2021)

[5] Humphreys, I.R., Pei, J., Baek, M., Krishnakumar, A., Anishchenko, I.V., Ovchinnikov, S., Zhang, J., Ness, T.J., Banjade, S., Bagde, S.R., Stancheva, V.G., Li, X., Liu, K., Zheng, Z., Barrero, D.J., Roy, U., Kuper, J., Fernández, I.S., Szakal, B., Branzei, D., Rizo, J., Kisker, C., Greene, E.C., Biggins, S., Keeney, S., Miller, E.A., Fromme, J.C., Hendrickson, T.L., Cong, Q., Baker, D.: Computed structures of core eukaryotic protein complexes. Science **374** (2021)

[6] Baek, M., DiMaio, F., Anishchenko, I., Dauparas, J., Ovchinnikov, S., Lee, G., Wang, J., Cong, Q., Kinch, L., Schaeffer, R., Millan, C., Park, H., Adams, C., Glassman, C., DeGiovanni, A., Pereira, J., Rodrigues, A., Dijk, A., Ebrecht, A., Opperman, D., Sagmeister, T., Buhlheller, C., Pavkov-Keller, T., Rathinaswamy, M., Dalwadi, U., Yip, C., Burke, J., Garcia, K., Grishin, N., Adams, P., Read, R., Baker, D.: Accurate prediction of protein structures and interactions using a 3-track neural network. Science (New York, N.Y.) **373**, 871–876 (2021)

[7] Zahn-Zabal, M., Michel, P.-A., Gateau, A., Nikitin, F., Schaeffer, M., Audot, E., Gaudet, P., Duek, P.D., Teixeira, D.D., Laval, V.R., Samarasinghe, K., Bairoch, A., Lane, L.: The nextprot knowledgebase in 2020: data, tools and usability improvements. Nucleic Acids Research **48**, 328–334 (2019)

[8] Lane, L., Argoud-Puy, G., Britan, A., Cusin, I., Duek, P.D., Evalet, O., Gateau, A., Gaudet, P., Gleizes, A., Masselot, A., Zwahlen, C., Bairoch, A.: nextprot: a knowledge platform for human proteins. Nucleic Acids Research **40**, 76–83 (2011)

[9] Paysan-Lafosse, T., Blum, M., Chuguransky, S., Grego, T., Pinto, B.L., Salazar, G.A., Bileschi, M.L., Bork, P., Bridge, A., Colwell, L., *et al.*: Interpro in 2022. Nucleic acids research **51**(D1), 418–427 (2023)

[10] Varga, J.K., Dobson, L., Tusnády, G.E.: Topdom: database of conservatively located domains and motifs in proteins. Bioinformatics **32**, 2725–2726 (2016)

[11] Quaglia, F., Mészáros, B., Salladini, E., Hatos, A., Pancsa, R., Chemes, L.B., Pajkos, M., Lazar, T., Peña-Díaz, S., Santos, J., Ács, V., Farahi, N., Fichó, E., Aspromonte, M.C., Bassot, C., Chasapi, A., Davey, N.E., Davidović, R., Dobson, L., Elofsson, A., Erdős, G., Gaudet, P., Giglio, M., Glavina, J., Iserte, J., Iglesias, V., Kálmán, Z., Lambrughi, M., Leonardi, E., Longhi, S., Macedo-Ribeiro, S., Maiani, E., Marchetti, J., Marino-Buslje, C., Mészáros, A., Monzon, A.M., Minervini, G., Nadendla, S., Nilsson, J.F., Novotný, M., Ouzounis, C.A., Palopoli, N., Papaleo, E., Pereira, P.J.B., Pozzati, G., Promponas, V.J., Pujols,

J., Rocha, A.C.S., Salas, M., Sawicki, L.R., Schad, E., Shenoy, A., Szaniszló, T., Tsirigos, K.D., Veljkovic, N., Parisi, G., Ventura, S., Dosztányi, Z., Tompa, P., Tosatto, S.C.E., Piovesan, D.: DisProt in 2022: improved quality and accessibility of protein intrinsic disorder annotation. Nucleic Acids Research **50**(D1), 480–487 (2022)

[12] Kumar, M., Gouw, M., Michael, S., Sámano-Sánchez, H., Pancsa, R., Glavina, J., Diakogianni, A., Valverde, J.A., Bukirova, D., Calyseva, J., Palopoli, N., Davey, N.E., Chemes, L.B.: Elm—the eukaryotic linear motif resource in 2020. Nucleic Acids Research **48**, 296–306 (2019)

[13] Hou, C., Wang, X., Xie, H., Chen, T., Zhu, P., Xu, X., You, K., Li, T.: PhaSepDB in 2022: annotating phase separation-related proteins with droplet states, co-phase separation partners and other experimental information. Nucleic Acids Research **51**(D1), 460–465 (2022)

[14] Mészáros, B., Erdős, G., Szabó, B., Schád, É., Tantos, Á., Abukhairan, R., Horváth, T., Murvai, N., Kovács, O.P., Kovács, M., *et al.*: Phasepro: the database of proteins driving liquid–liquid phase separation. Nucleic acids research **48**(D1), 360–367 (2020)

[15] Käll, L., Krogh, A., Sonnhammer, E.L.L.: A combined transmembrane topology and signal peptide prediction method. Journal of molecular biology **338 5**, 1027–36 (2004)

[16] Erdös, G., Pajkos, M., Dosztányi, Z.: Iupred3: prediction of protein disorder enhanced with unambiguous experimental annotation and visualization of evolutionary conservation. Nucleic Acids Research **49**, 297–303 (2021)
